# MRI radiomics for early prediction of response to vaccine therapy in a transgenic mouse model of pancreatic ductal adenocarcinoma

**DOI:** 10.1186/s12967-020-02246-7

**Published:** 2020-02-10

**Authors:** Aydin Eresen, Jia Yang, Junjie Shangguan, Yu Li, Su Hu, Chong Sun, Yury Velichko, Vahid Yaghmai, Al B. Benson, Zhuoli Zhang

**Affiliations:** 1 Dept. of Radiology, Feinberg School of Medicine, Northwestern University, 737 N. Michigan Ave, Suite 1600, Chicago, IL 60611 USA; 2 Dept. of Gastrointestinal Surgery, Affiliated Hospital of Qingdao University, Qingdao, Shandong China; 3 Dept. of Radiology, First Affiliated Hospital of Soochow University, Changzhou, Jiangsu China; 4 Dept. of Orthopaedics, The Affiliated Hospital of Qingdao University, Qingdao, Shandong China; 5 Robert H. Lurie Comprehensive Cancer Center of Northwestern University, 676 N. St. Clair, Suite 850, Chicago, IL 60611 USA; 6 Dept. of Radiological Sciences, School of Medicine, University of California, Irvine, CA USA; 7 Division of Hematology and Oncology, Feinberg School of Medicine, Northwestern University, Chicago, IL USA

**Keywords:** Dendritic cell vaccine, Machine learning, Magnetic resonance imaging, Pancreatic ductal adenocarcinoma, Radiomics

## Abstract

**Background:**

There is a lack of well-established clinical tools for predicting dendritic cell (DC) vaccination response of pancreatic ductal adenocarcinoma (PDAC). DC vaccine treatment efficiency was demonstrated using histological analysis in pre-clinical studies; however, its usage was limited due to invasiveness. In this study, we aimed to investigate the potential of MRI texture features for detection of early immunotherapeutic response as well as overall survival (OS) of PDAC subjects following dendritic cell (DC) vaccine treatment in *LSL*-*Kras*^*G12D*^*;LSL*-*Trp53*^*R172H*^*;Pdx*-*1*-*Cre* (KPC) transgenic mouse model of pancreatic ductal adenocarcinoma (PDAC).

**Materials and methods:**

KPC mice were treated with DC vaccines, and tumor growth was dynamically monitored. A total of a hundred and fifty-two image features of T2-weighted MRI images were analyzed using a kernel-based support vector machine model to detect treatment effects following the first and third weeks of the treatment. Moreover, univariate analysis was performed to describe the association between MRI texture and survival of KPC mice as well as histological tumor biomarkers.

**Results:**

OS for mice in the treatment group was 54.8 ± 22.54 days while the control group had 35.39 ± 17.17 days. A subset of three MRI features distinguished treatment effects starting from the first week with increasing accuracy throughout the treatment (75% to 94%). Besides, we observed that short-run emphasis of approximate wavelet coefficients had a positive correlation with the survival of the KPC mice (*r *= 0.78, *p *< 0.001). Additionally, tissue-specific MRI texture features showed positive association with fibrosis percentage (*r *= 0.84, *p *< 0.002), CK19 positive percentage (*r *= − 0.97, *p *< 0.001), and Ki67 positive cells (*r *= 0.81, *p *< 0.02) as histological disease biomarkers.

**Conclusion:**

Our results demonstrate that MRI texture features can be used as imaging biomarkers for early detection of therapeutic response following DC vaccination in the KPC mouse model of PDAC. Besides, MRI texture can be utilized to characterize tumor microenvironment reflected with histology analysis.

## Background

Pancreatic ductal adenocarcinoma (PDAC) is the 3rd most common cause of cancer-related deaths in the United States with an expected 44,330 deaths in 2018 [1]. Due to the early local and metastatic spread of the cancerous cells, almost 80-90% of the patients are unsuitable for complete surgical resection [2]. Even after successful surgery, the recurrence of PDAC is excessively common; therefore, 5-year survival remains at a lower rate of 6% in the United States [2]. This introduces a common interest to develop novel treatments and determine quantitative MRI characteristics that can serve as PDAC survival biomarkers and are correlated with the gold standard and histopathological outcomes [3].

Among all the treatment methods, immunotherapy has become one of the most promising approaches for PDAC in recent years [4]. Dendritic cell (DC) is the main antigen-presenting cell of the immune system [5] and a key mediator of tumor immunity owing to its unique capacity for cross-presenting tumor-associated antigens to CD8^+^ T cells in the draining lymph nodes [6], providing a rationale for their utilization as cancer vaccines. Recent clinical trials have demonstrated that DC-based cancer vaccines can efficiently induce tumor-specific effector T cells in patients with PDAC [7].

The biological effects of immunotherapies can be identified using histological analysis that reveals the characteristics of the tissues on a cellular level. Molecular imaging characteristics obtained with histological processes (cellular proliferation and structural integrity) may correlate with poor prognosis of pancreatic cancer tissues [8, 9]. Specifically, the cell proliferation marker Ki67 protein expressed by *MKI67* is correlated with high-risk prognosis described by increased recurrence and decreased overall survival [10]. Besides, cytokeratin 19 (ductal marker) encoded by the *KRT19* gene has been described to show strong immunoreactivity in PDAC tumors [11] that could be utilized to interpret the treatment effects in PDAC.

Although molecular imaging provides the cellular level characteristics of tissues, radiological imaging modalities such as MRI is preferred due to noninvasive analysis of the tissues and smooth translation to clinical practice [12, 13]. However, cancer immunotherapy can cause unique response patterns such as a transient increase in tumor size (pseudoprogression), delayed or subsequent regression, and the existence of new lesions; hence, traditional size-based solid tumor response criteria (RECIST 1.1) may be inappropriate for evaluation of the therapeutic response [14]. Therefore, advanced approaches utilizing characteristics of the tumor tissues are needed to detect the therapeutic response.

In recent years, texture analysis has become a popular approach for quantitative analysis of the malignancies in biomedical studies [15–17]. In texture analysis, the characteristics of the underlying structures were described by high-throughput features associated with texture or complex patterns of the tissues [16]. Previous studies investigated the performance of the texture analysis approach in terms of accurate prediction of patient survival, diagnosis, and detection of treatment response using clinical data [18–21]. However, response to immunotherapy has not been studied with the assessment of histopathological analysis for validation of the outcomes. Hence, preclinical studies are required to evaluate the potential of the texture analysis approach to determine early therapeutic response and long-term outcomes following immunotherapy.

In this study, we aimed to identify therapeutic responses after DC vaccine treatment in the KPC mouse model of PDAC using MRI texture features, characterize tumor tissue by describing the association between MRI and histological tumor markers and assessing the long-term outcome of PDAC subjects.

## Materials and methods

**Fig. 1:**
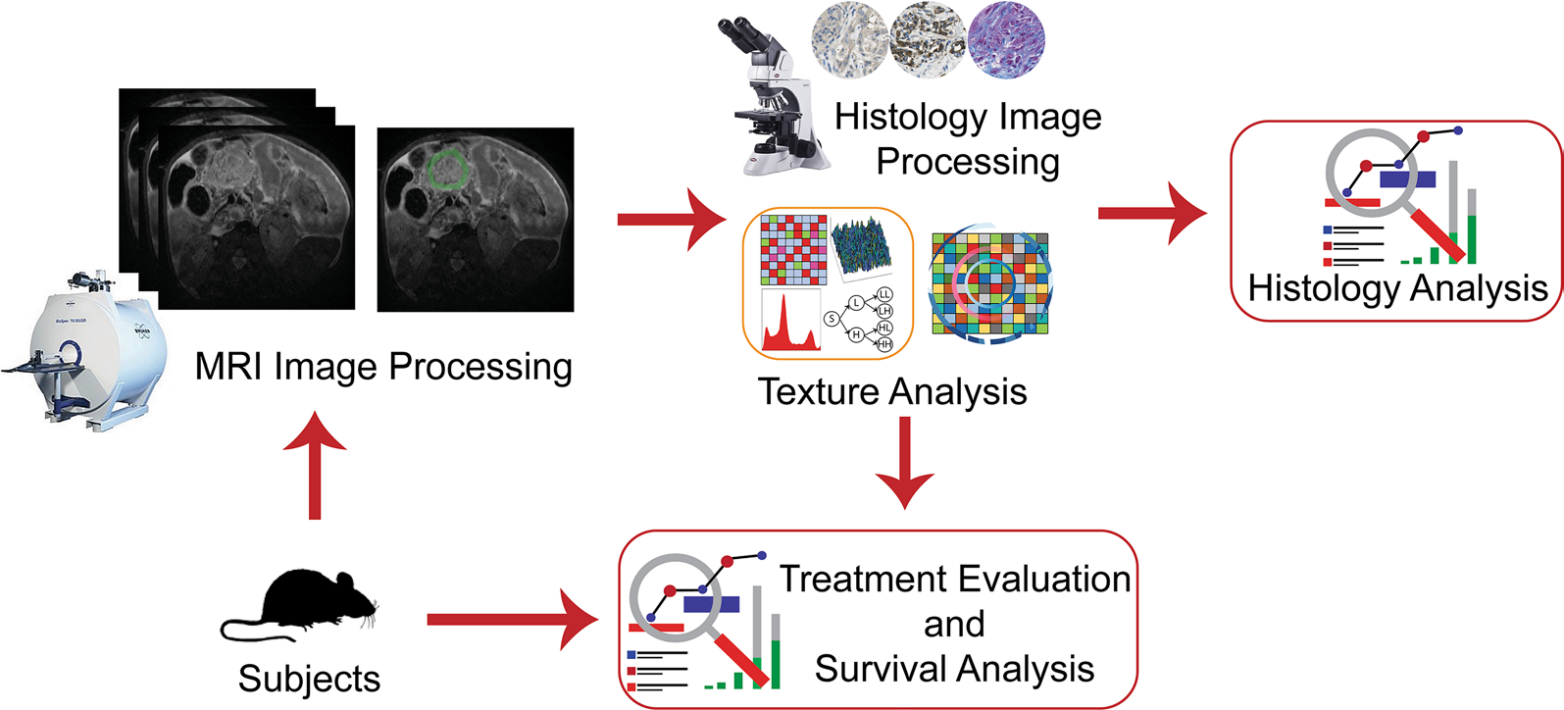
The framework of the study representing the steps. Histology analysis focuses on identifying the association between pathological outcomes and radiomic features. In treatment evaluation, the quantitative MRI features were utilized to distinguish treated and untreated tumor tissue. Survival analysis includes the investigation of the correlation between MRI features and survival of PDAC mice

Our pre-clinical animal study was approved by the Institutional Animal Care and Use Committee of Northwestern University, and the experiments were conducted according to the guidelines for laboratory animal use [22]. The flow diagram of our study was presented in Fig. 1 to describe completed experiments during our study.

### Animal model and treatment of tumor

The *LSL*-*Kras*^*G12D/*+^; *LSL*-*Trp53*^*R172H/*+^; *Pdx*-*1*-*Cre* (KPC) mouse model demonstrates high similarity in pathophysiological aspects and reflects biological features of human PDAC [23]; therefore, this model was commonly used in preclinical studies as well as in our study. The mouse strains *p53*^*LSL*−*R270H*^ (strain number 01XM3), *Kras*^*LSL*−*G12D*^ (strain number 01XJ6), and *Pdx*-*1*-*Cre* (strain number 01XL5) were purchased from the Jackson Laboratory (Bar Harbor, ME), and KPC mice were generated in our laboratory following previously described approach [24]. A total of sixteen KPC mice were bred for our study and randomly separated into two groups e.g. untreated control group and DC vaccine treatment group.

When KPC mice reached 3 months of age, we started to scan each mouse biweekly using a small animal MRI scanner to monitor the progression of the tumor. When tumor size reached 0.2–0.5 cm in diameter, the KPC mice were enrolled in this study. The mice in the treatment group received 3 × 10^6^ DCs pulsed with irradiated KPC cell by intraperitoneal administration (IP) injection weekly for 3 weeks after enrollment. Survival events were scored when mice lost more than 15% of the body weight, tumor diameter became larger than 1.8 cm, decreased mobility, extreme lethargy, or per absolute survival event. All enrolled mice were weighed and checked for signs of distress regularly during the study.

### MRI acquisition and processing

A 7.0T Bruker small-bore pre-clinical scanner (Clinscan, Ettlingen, Germany) with a commercial coil (Clinscan, Ettlingen, Germany) was used to acquire T2-weighted MRI data of the KPC mice. We scanned each mouse weekly during the treatment period to evaluate tumor progression. During MRI scans, the mice were under anesthesia provided by an automatic delivery system (Isoflurane Vaporizer, Rockmart, GA) and restrained in the supine position. The body temperature was adjusted using a water-bed heating system (SA Instruments, Stony Brook, NY), and the respiratory rate of mice was monitored to trigger an MRI sequence. The pancreatic tumor was located using 10 slices of coronal T2-weighted images (TR/TE: 1600/37 ms; slice thickness: 1.0 mm; flip angle: 180°; a field of view: 36 mm × 28 mm). Later, we acquired transverse T2-weighted MRI images with fat suppression sequence (TR/TE: 2100/40 ms; slice thickness: 0.5 mm; flip angle: 180°; a field of view: 21 mm × 30 mm).

The tumor tissue regions were identified and a region of interest (ROI) was drawn on the slice with maximal tumor diameter by an expert radiologist with 5 years of experience, and then second expert radiologist with more than 10 years of experience inspected and validated the segmentation of tumor tissues for each mouse. The intensity of the ROIs was then normalized and scaled to the same range for each subject prior to feature extraction.

### Histology image acquisition and processing

After animals reached experimental endpoints [25], they were sacrificed according to the regulation and pancreatic tissues were harvested as a whole during the surgery. PDAC tumors were sliced, fixed in 10% formalin, and embedded in paraffin. 5 μm sections of paraffin-embedded pancreatic tissues were analyzed for H&E and Masson’s Trichrome dyes according to the instructions of the manufacturers. For immunohistochemistry analysis, 5 μm thick sections were deparaffinized in xylene, rehydrated in graded ethanol and subjected to antigen retrieval by steam heating in Citra antigen retrieval solution (Vector). After blocking for 1 h at room temperature in blocking buffer (5% goat serum, 2.5% BSA in 1 × PBS), slides were incubated overnight in a humidified chamber at 4 °C with anti-mouse CK19, rabbit monoclonal anti-Ki67 (clone SP6, Invitrogen) and rat monoclonal anti-mouse CD8 (Clone 4SM15, Invitrogen). Immunostaining was detected using 3,3′-diaminobenzidine (DAB) (Vector). Whole tissue slide scans were performed on the TissueFAXS system and quantitative analysis was completed using ImageJ at the maximum magnification of acquired images [26].

### Feature extraction

Feature extraction is the initial step of the texture analysis framework where a high-dimensional data structure portraying distinct characteristics of biological tissues is constructed to distinguish differences or relationships among multi forms of imaging data. In this study, we analyzed various characteristics including gray-level intensity distribution, texture, pattern, gradient and shape of the MRI images to capture tissue-specific information reflecting the outcomes of DC treatment. Imaging biomarkers of pancreatic tumor of KPC mice were extracted utilizing seven feature families-first-order statistics (FoS), gray-level co-occurrence matrix (GLCM), gray-level run-length matrix (GLRM), local binary patterns (LBP), fractal analysis (FA), histogram of oriented gradients (HoG), and shape- with two filters -gradient and wavelet [27, 28]. After generating wavelet images using Haar basis functions, we extracted power, FoS, GLCM, and GLRM features of four sub-wavelet images. Additionally, we calculated FoS features of HoG resulting in a hundred and fifty-two features of T2-weighted MRI images.

### Statistical analysis

To identify the potential of MRI image features for distinguishing the treatment effects, we assessed all the features after evaluation of the cross-correlation of the features and then removed the variables with strong correlations. The remaining features were utilized to build a kernel-based support machine classifier with a leave-one-out validation approach using an exhaustive search feature selection method. The number of features was increased while generating binary classifiers until the performance of the model did not improve more than a pre-selected threshold value (1E−3).

The individual image features were evaluated using univariate analysis with the Pearson correlation coefficient. The significant features with strong correlation were identified as MRI texture biomarkers for the survival of PDAC subjects. Additionally, we investigated the potential of image features to predict histological tumor markers obtained with pathological analyses. Pearson correlation was employed to identify associated features with histological tumor markers using a two-tailed Student T-test by evaluating the significance of MRI texture features. The individual features that demonstrated a strong correlation with the histological biomarkers were identified as non-invasive MRI imaging markers for disease evaluation. All the statistical analyses were performed using Matlab^®^ v.9.5.0 (MathWorks, MA) and *p *< 0.05 was accepted as statistically significant.

## Results

**Fig. 2:**
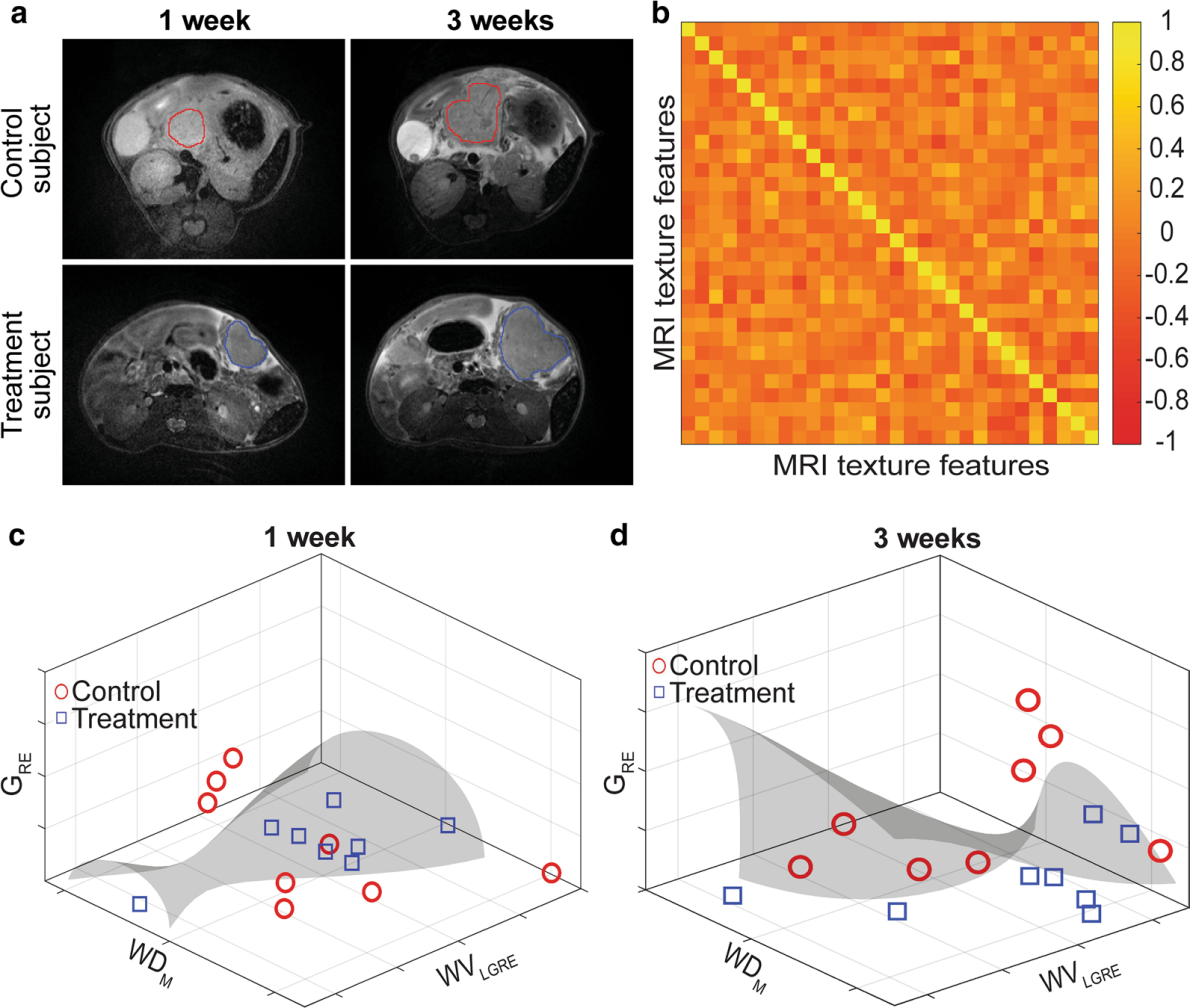
The treatment effect observed on qualitative and qualitative MRI analyses. **a** Representative T2W images showing the tumor growth for mice in control and treatment groups. **b** Heatmap demonstrating the association among the features after reduction. **c** The treated tumor was differentiated from the untreated tumor at the 1st and 3rd weeks of the treatment. G_RE_: Long run emphasis of the gradient image. WD_M_: Mean of diagonal wavelet coefficients. WV_LGRE_: Low gray-level emphasis of vertical wavelet coefficients

A total of sixteen KPC mice were randomly separated into control and treatment groups when tumors were detectable by MRI (0.2–0.5 cm in the longest diameter). The survival characteristics of the individuals in each group were monitored regularly. There was no statistically significant difference in tumor size between KPC mice in untreated control and DC vaccine treatment groups during the first three weeks of the study (*p*_1-week_ = 0.7161, *p*_3-week_ = 0.4465). A sample from each group was presented in Fig. 2a.

A total of a hundred and fifty-two features was extracted using seven feature extraction methods and two filters. Following the removal of highly correlated (*r *> 0.50) features, we had thirty features including FoS (2), gradient (4), GLRM (1), HoG (2), shape (3), and wavelet (18) to distinguish the treatment effects using T2-weighted MRI data. The correlations between the selected features for the detection of treatment effects were demonstrated in Fig. 2b. By performing an exhaustive assessment of the MRI texture features, a set of three features (mean of diagonal wavelet coefficients, the low gray-level run emphasis of vertical wavelet coefficients and long-run emphasis of gradient image) were identified according to the performance of the generated kernel-based support vector machines model. The generated classifier identified the treated tumors with an increasing accuracy from 75% to 93.75% throughout the first three weeks of the DC vaccine treatment. In Fig. 2c and d, we visualized the classification of the untreated and treated KPC mice using the support vector machines model that generated using the same MRI texture features.

**Fig. 3:**
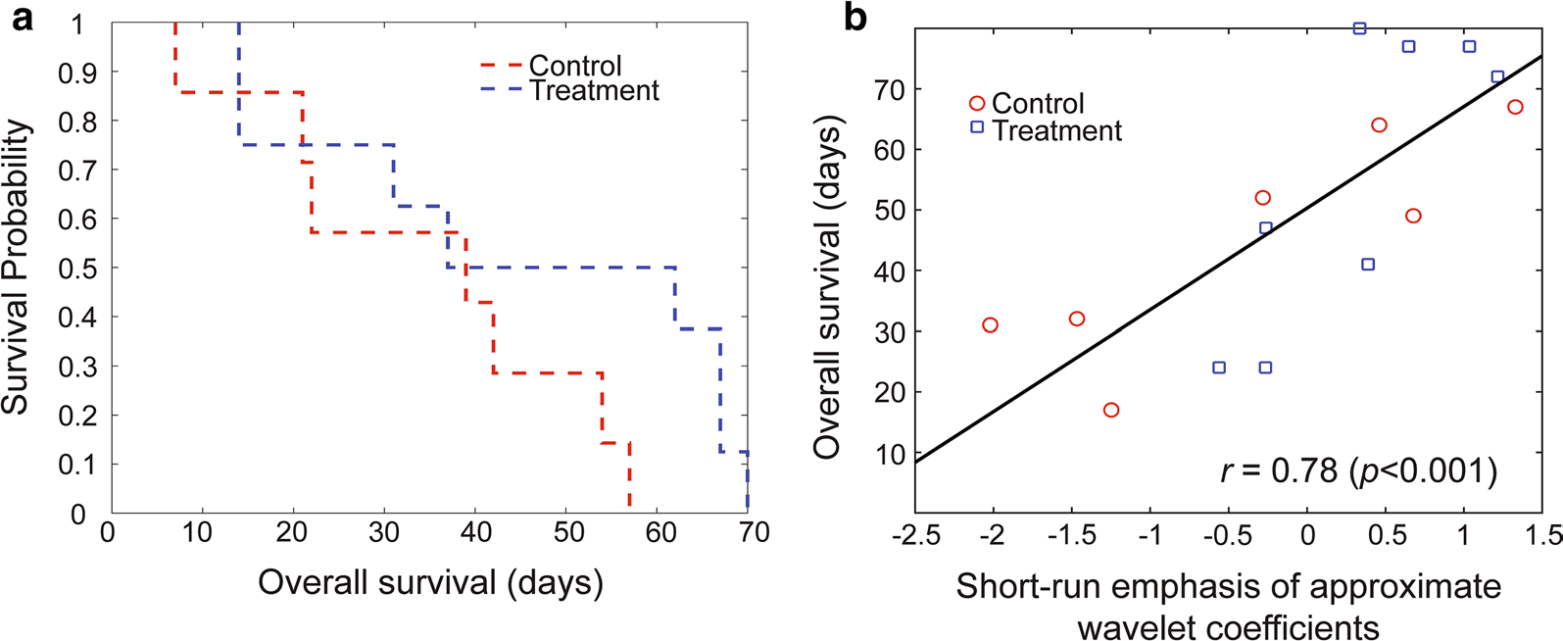
The overall survival of the KPC mice in untreated control and DC vaccine treatment groups. The survival of the KPC mice was demonstrated according to the Kaplan–Meier method. An MRI texture feature demonstrated a strong correlation with the survival of the subjects

The survival of the KPC mice in untreated control and DC vaccine groups were also evaluated using the Kaplan–Meier method (Fig. 3a). Although there was not a significant difference between untreated control and treated KPC mice (*p* = 0.35), the survival of the treated mice (54.8 ± 22.54 days) was better than untreated control mice (35.39 ± 17.17 days). Furthermore, we evaluated the relationship between individual MRI texture features and survival of the KPC mice regardless of the treatment status by measuring the Pearson correlation coefficient in univariate analysis to describe the long-term behavior of the KPC mice. The short-run emphasis of approximate wavelet coefficients demonstrated a strong correlation with the survival of the KPC mice (*r* = 0.78, *p* < 0.001). In Fig. 3b, we visualized the survival of the KPC mice in untreated control and DC vaccine treatment groups with respect to identified MRI texture feature.

**Fig. 4:**
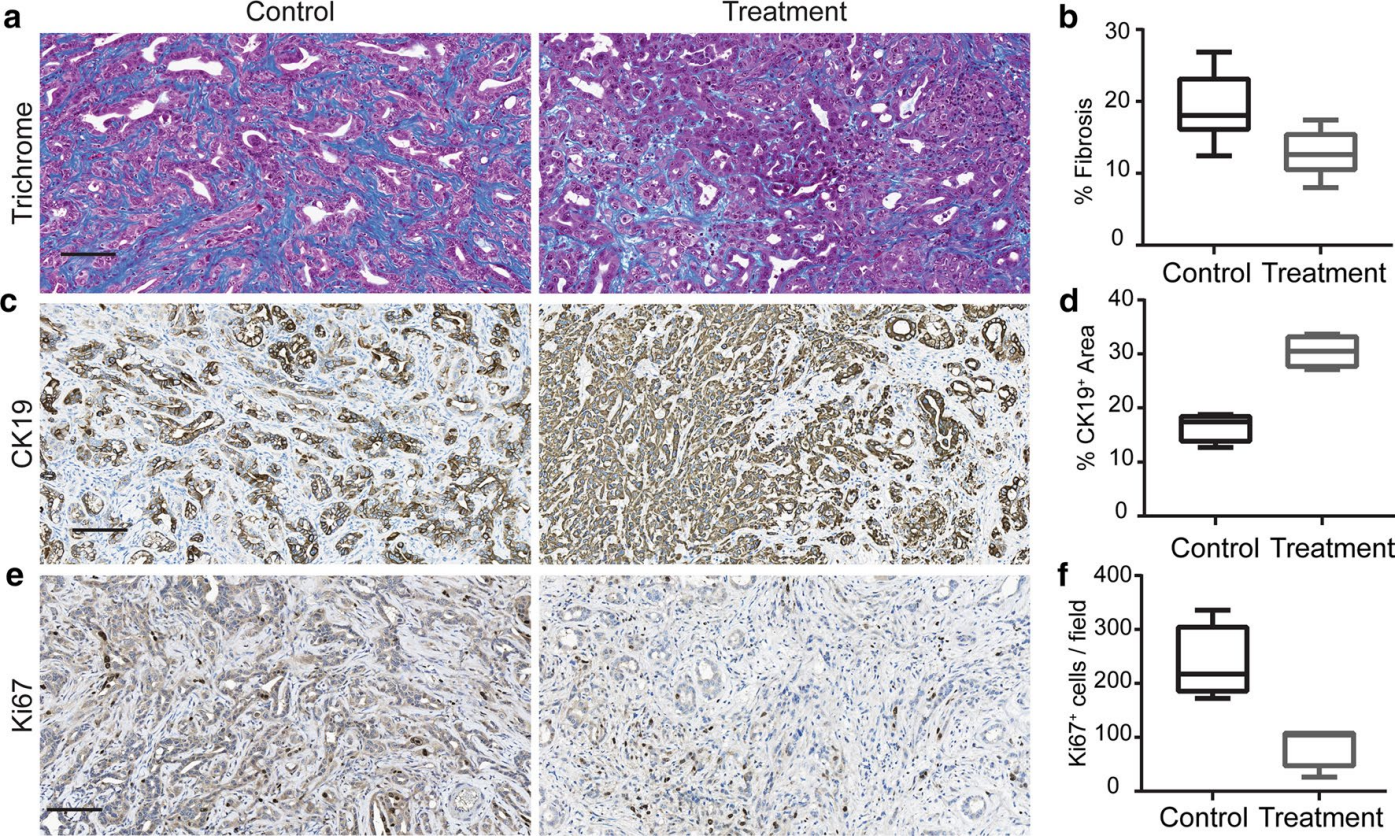
The qualitative and quantitative histological analysis. **a** describes the biological difference between treated and control mice on histology images stained with trichrome, CK19 and Ki67 dyes. **b** Summarizes the statistics of histological biomarkers

In the histological analysis of the sample tissues, we observed that KPC mice in the treatment group had significantly decreased levels of collagen deposition throughout the pancreas compared with mice in the control group (Fig. 4a, b). Moreover, CK19 (ductal marker) percentage was larger in the treatment group than in the control group (Fig. 4c, d), and pancreatic cell proliferation assessed by Ki67 immunostaining was lower in the treatment group than in the control group (Fig. 4e, f). Taken together, these data demonstrated that the DC vaccine treatment can effectively suppress PDAC tumor progression in KPC mice.

**Fig. 5:**
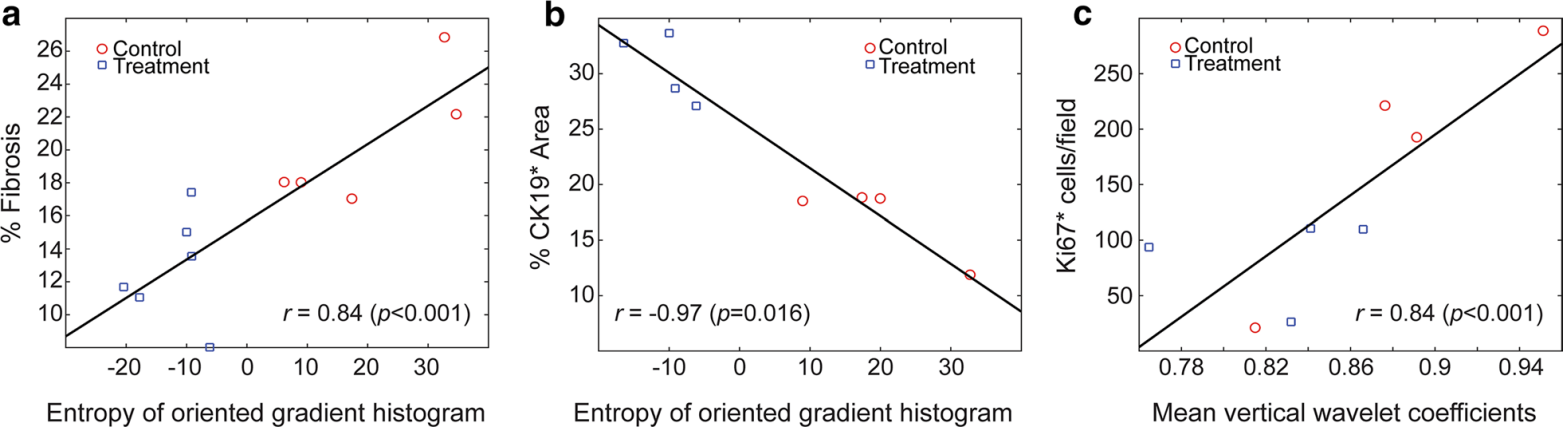
T2-weighted MRI image features have demonstrated a strong correlation with histological tumor biomarkers

For evaluation of texture analysis in association with pathological findings as to the gold standard of disease analysis, we employed a two-tailed Student T-test to determine MRI texture features that remarkably characterize the histological outcomes. For trichrome staining histology images, three image features identified with Student T-test listed in Table 1, showed strong correlation with fibrosis percentages (*r *= [0.84, 0.60, 0.64], *p* = [0.001, 0.049, 0.03]), individually. The behavior of the fibrosis percentages with respect to an MRI texture feature (the entropy of the oriented gradient histogram) was described in Fig. 5a. Besides, we also investigated the association between MRI texture and tumor biomarkers e.g. CK19 and Ki67 immunostains. During our experiments, we observed that three features listed in Table 1 selected according to their T values highly correlated with percentage of CK19 positive region (*r *= [− 0.97, 0.76, − 0.84], *p* = [0.0001, 0.03, 0.009]). The characteristics of tissues of treated and control mice were presented in Fig. 5b with respect to the entropy of the oriented gradient histogram of T2-weighted MRI data. Furthermore, we observed a strong correlation between the mean vertical wavelet coefficients of the T2-weighted MRI texture and the number of Ki67 cells/field proposing the potential of texture characteristic to predict Ki67 related proliferation (Fig. 5c).

**Table 1:** List of features correlated with histological analysis as comparing the effects of treatment

Features	Control group Mean ± S.D.	Treatment group Mean ± S.D.	*p*	Cohen’s d
Trichrome				
Entropy of histogram of gradients	19.98 ± 13.2	− 12.10 ± 5.63	0.0007	3.162
Long run emphasis of diagonal wavelet coefficients	0.64 ± 0.09	0.47 ± 0.05	0.003	2.473
Skewness of vertical wavelet coefficients	0.92 ± 0.01	0.89 ± 0.02	0.001	1.827
CK19				
Entropy of histogram of gradient	19.77 ± 9.84	− 10.42 ± 4.33	0.0014	3.971
Variance of diagonal wavelet coefficients	0.06 ± 0.02	0.18 ± 0.08	0.018	2.058
Long run emphasis of vertical wavelet coefficients	0.52 ± 0.13	0.30 ± 0.05	0.019	2.233
Ki67				
Mean of vertical wavelet coefficients	0.88 ± 0.0560	0.83 ± 0.0433	0.155	1.151

## Discussion

Our study demonstrated that MRI texture features can serve as imaging biomarkers for early detection of immunotherapeutic response after DC vaccine treatment as well as the long-term outcomes in the KPC mouse model of PDAC.

The quantitative image features extracted from T2-weighted MRI data can be used to measure the characteristics of tumors in the KPC mouse model. The determined set of three image features distinguished DC vaccinated tumors from untreated tumor tissue starting from the first week of the treatment (75%). The classification accuracy reached 93.75% in the third week of the treatment. Besides, an MRI texture feature was identified during univariate analysis to predict the survival of the KPC mice in control and DC vaccine treatment groups. Additionally, we used noninvasive MRI texture features to describe the molecular characteristics of the tumor tissue obtained with invasive histopathological analysis.

The prognostic potential of quantitative imaging features for PDAC in the analysis was reported by several studies [29–32]. In an earlier study, twenty-six PDAC subjects were studied to identify clinical or textural features associated with survival by comparing the change between pre- and post-treatment ^18^F-FDG-PET/CT images [32]. The identified features were provided as a feasible approach for evaluation and prediction of clinical outcomes. A recent study focused on T2-weighted MRI images to assess the association between radiomics features and pathological aggressiveness or clinical outcomes of 18.5 months after pancreatectomy procedure [30]. As tumor size was observed as a significant element to predict recurrence-free and overall survival, entropy with medium texture analysis was associated with the survival of PDAC patients. Moreover, radiomic analyses of T2-weighted and diffusion-weighted MRI images were implemented to identify therapy-dependent variations in tumor biology of PDAC in preclinical animal studies [31]. The random forest classifier model with radiomic features extracted from T2-weighted and ADC map images was able to differentiate tumor variation after the treatment. The results suggest that imaging biomarkers based on radiomic features are potential tools for the evaluation of tumor responses.

In our study, we focused on the investigation of the radiomic analysis method to early detect responses to the tumor tissue changes to DC vaccination. A set of three radiomic features differentiated DC vaccine treatment effects with increasing accuracy during the treatment period (Fig. 2c and d); no statistically significant difference in tumor size was found. Besides, a substantial association between quantitative MRI features and survival of the KPC mice was observed (Fig. 3) that demonstrated the potential of characteristics of MRI texture to interpret long-term outcomes of the patients with PDAC disease. Furthermore, we described the association between MRI texture feature and histological disease biomarkers of cancerous tissue e.g. fibrosis percentage (*r *= 0.84, *p* < 0.001), CK19 positive area (*r *= − 0.97, *p* < 0.001) and Ki67 positive cell percentage (*r *= 0.81, *p* = 0.016). The selected quantitative imaging features extracted from T2-weighted MRI data were chosen to detect structural changes or patterns of heterogeneity in tumor tissue that occurred during the DC vaccine treatment. These changes were identified at the cellular level using histological biomarkers, and changes in tissue characteristics were also identified using a univariate model generated with texture features of MRI data. Taken together, the results demonstrate that MRI texture provides promising knowledge to describe pancreatic tumor characteristics and detect early treatment, as verified by gold standard biological analysis.

There were several limitations to our study. First, tumors were segmented using a manual approach which may introduce user bias and requires additional processing time; however, it is a commonly used approach in pre-clinical studies. Implementing an automated approach for tumor segmentation would allow studies with larger cohorts by reducing potential user bias and time consumption. Second, there was a low number of subjects in our experiments although it’s comparable to other pre-clinical studies. The prognostic histological marker Ki67 was significantly decreased in the treated group while no significant difference in survival between untreated control and treated KPC mice. This may be particularly relevant to the limited number of subjects in each group. The number of subjects will be eventually increased with further studies. Finally, we only considered individual treatment methods to monitor the effects of tumor progression and pathological analysis. This could be addressed by collaborating with multiple institutions.

## Conclusions

MRI texture features can be used as noninvasive imaging biomarkers to early predict therapeutic responses of DC vaccination in addition to the overall survival of the subjects in the KPC mouse model of PDAC.

## Data Availability

The datasets generated during and/or analyzed during the current study are available from the corresponding author on reasonable request.

## Abbreviations

DC: Dendritic cell
FoS: First order statistics
GLCM: Gray-level co-occurrence matrix
GLRM: Gray-level run-length matrix
LBP: Local binary patterns
FD: Fractal dimension
HoG: Histogram of oriented gradients
KPC: LSL-Kras^G12D^; LSL-Trp53^R172H^; Pdx-1-Cre
PDAC: Pancreatic ductal adenocarcinoma
ROI: Region of interest
